# 罗沙司他治疗难治性非重型再生障碍性贫血的疗效与安全性

**DOI:** 10.3760/cma.j.cn121090-20230902-00101

**Published:** 2024-03

**Authors:** 璐 徐, 青林 胡, 辰 杨, 苗 陈, 冰 韩

**Affiliations:** 1 北京协和医院血液内科，北京 100730 Department of Hematology, Peking Union Medical College Hospital, Chinese Academy of Medical Sciences & Peking Union Medical College, Beijing 100730, China; 2 海南医学院第一附属医院血液内科，海口 570102 Department of Hematology, The First Affiliated Hospital of Hainan Medical University, Haikou 570102, China

**Keywords:** 罗沙司他, 非重型再生障碍性贫血, 难治性, 疗效, 安全性, Roxadustat, Non-severe aplastic anemia, Refractory, Efficacy, Safety

## Abstract

**目的:**

评估罗沙司他在难治性非重型再生障碍性贫血（NSAA）患者中的疗效与安全性。

**方法:**

回顾性收集2020年10月至2022年8月在北京协和医院连续使用罗沙司他至少3个月，并在使用后随访超过6个月的难治性NSAA患者的临床资料，收集其人口学信息、临床资料、疗效、不良反应及转归，并分析可能影响疗效的因素。

**结果:**

共纳入41例患者。男女比例为16∶25，中位年龄52（18～84）岁，罗沙司他中位治疗时间5（3～20）个月，中位随访时间15（6～26）个月。1、2、3、6、12个月的血液学改善-红系（HI-E）率分别为12.2％、29.3％、46.3％、43.9％和30.3％。治疗后3、6、12个月摆脱输血依赖比例分别为28.5％、38.1％、33.3％。治疗后部分患者HGB恢复正常。不良事件发生率为22％，均为Ⅰ～Ⅱ级，可恢复。未发现影响HI-E的因素。至随访期末，有45％（9/20）的患者复发，中位复发时间为7（3～12）个月。未观察到克隆演变。1例患者死于肺部感染。

**结论:**

罗沙司他可有效改善难治性NSAA的贫血，安全性好。

## Abstract (en)

**Objective:**

To evaluate the efficacy and safety of roxadustat in patients with refractory non-severe aplastic anemia（NSAA）.

**Methods:**

The clinical data of patients with refractory NSAA who had been treated with roxadustat continuously for at least 3 months and followed up for more than 6 months at Peking Union Medical College Hospital from October 2020 to August 2022 were retrospectively collected. The demographic information, clinical data, treatment efficacy, adverse reactions, and outcomes were evaluated, and the factors influencing efficacy were analyzed.

**Results:**

A total of 41 patients were included. The male-to-female ratio was 16∶25, and the median age was 52（18–84）years. The median duration of roxadustat treatment was 5（3–20）months, and the median follow-up was 15（6–26）months. Hematologic improvement-erythroid（HI-E）was 12.2%, 29.3%, 46.3%, 43.9%, and 30.3% at 1, 2, 3, 6, and 12 months, respectively. The rate of transfusion independence was 28.5%, 38.1%, and 33.3% at 3, 6, and 12 months, respectively. Hemoglobin returned to normal in some patients after treatment with roxadustat. The incidence of adverse events was 22%, all of which were grade Ⅰ–Ⅱ and recoverable. No factors that could affect HI-E were identified. By the end of follow-up, 45% of the patients relapsed, with a median time to relapse of 7（3–12）months. No clonal evolution was observed, and one patient died.

**Conclusion:**

Roxadustat effectively improved anemia with good tolerance in patients with refractory NSAA.

再生障碍性贫血（AA）是一种骨髓造血衰竭综合征。其发病率在我国约为0.74/10万，可发生于各年龄段，以老年人更为多见。按疾病严重程度，分为重型AA（SAA）和非重型AA（NSAA）。SAA的一线治疗方案主要是以抗胸腺细胞球蛋白（ATG）和环孢素 A（CsA）为基础的免疫抑制治疗（IST）与造血干细胞移植（HSCT）[1]–[2]。输血依赖NSAA治疗原则同SAA，但由于ATG有一定治疗风险，国内多采用以CsA为主的治疗[3]–[4]。目前IST的治疗反应率仅为40％～70％[5]，部分患者经过足量、足疗程的CsA治疗，依然无法获得任何血液学改善；即使在有治疗反应的患者中，部分患者仍无法达到完全缓解（CR）；而HSCT则存在供者寻找困难，移植相关并发症多等问题[6]。

罗沙司他是一种新型口服的小分子药物，通过抑制低氧诱导因子（HIF）脯氨酰羟化酶来防止HIF-1α的降解[7]–[8]。HIF蛋白主要激活促红细胞生成素（EPO），EPO的释放导致骨髓中红细胞的产生。此外，罗沙司他还可以下调铁调素水平，改善铁的吸收和利用障碍[9]，从而改善造血。罗沙司他于2018年12月获得国家药品监督管理局批准，用于慢性肾脏病透析患者的贫血治疗[10]。目前罗沙司他已进行多个临床试验，集中在肾性贫血的治疗[7],[9],[11]–[13]。2021年Henry等[14]研究发现罗沙司他可改善低危骨髓增生异常综合征（MDS）患者的贫血症状，提高血红蛋白水平（NCT03263091）。此外，罗沙司他在肿瘤化疗相关性贫血的研究也已接近尾声（NCT04076943）[15]。

基于罗沙司他在多种贫血相关疾病可能有效的情况，我们中心尝试使用罗沙司他治疗难治性NSAA患者，现将其疗效及安全性报道如下。

## 病例与方法

一、病例资料

本研究为回顾性观察性研究。收集2020年10月至2022年8月在北京协和医院门诊明确诊断难治性NSAA并应用罗沙司他治疗的病例资料。AA诊断及分型符合《再生障碍性贫血诊断治疗专家共识（2022年版）》标准[1]。为避免前期治疗差异带来的影响，本研究排除了既往接受过ATG或HSCT的患者。难治定义为至少经过包含足量CsA（3～5 mg/kg）治疗至少6个月，且评价为无效者；PNH克隆阳性定义为流式细胞术CD24^+^/Flaer^−^粒细胞比例>1％（10 000个有核细胞）。

纳入标准：①确诊为NSAA；②年龄≥18岁；③符合难治性AA定义；④在开始罗沙司他之前已停止其他治疗至少3个月；⑤罗沙司他治疗至少3个月；⑥罗沙司他治疗后至少随访6个月；⑦基线血清肌酐（Scr）≤1.5正常值上限（ULN）。本研究排除了在使用罗沙司他前患有活动性感染，活动性结缔组织疾病，严重肝脏、肾脏或心脏疾病的患者。本研究经北京协和医院伦理委员会批准（批件号：1-23PJ1532）。

收集51例患者病例资料，6例因服药时间不足，4例因随访时间不足而被剔除，最终纳入41例患者进行分析。

二、治疗方案

罗沙司他剂量为2.5 mg·kg^−1^·d^−1^，隔日1次，治疗后每月复查，HGB连续2个月≥120 g/L或出现严重不良反应停药，否则至少使用3个月以上，如果6个月仍无任何改善，可以停药。ANC<0.5×10^9^/L时可使用G-CSF，直至ANC≥1.0×10^9^/L。HGB<60 g/L时可以输注红细胞，PLT<10×10^9^/L时可以输注血小板，治疗后出现感染者予对症治疗。无其他合并用药。

三、疗效评价标准及相关定义

治疗的血液学反应主要通过外周血红细胞、粒细胞、血小板反应评估。疗效判定标准为：①CR：HGB>100 g/L、PLT>100×10^9^/L及ANC>1.5×10^9^/L。②部分缓解（PR）：至少一系达正常水平或倍增，或初始HGB<60 g/L经治疗后至少升高30 g/L；或初始ANC<0.5×10^9^/L经治疗后至少升高0.5×10^9^/L；或初始PLT<20×10^9^/L经治疗后至少升高20×10^9^/L。③未缓解（NR）：血常规下降或未达PR标准。参考国际标准定义工作组（IWG）2006年的标准[16]，血液学改善-红系（HI-E）定义为每8周基线输血负担≥ 4个单位的患者，每8周减少红细胞输注≥4个单位；每8周基线输血负担<4个单位的患者，HGB水平增加≥15 g/L。血液学改善-粒系（HI-N）定义为ANC升高>0.5×10^9^/L。血液学改善-血小板（HI-P）定义为血小板升高，基线PLT≥20×10^9^/L时，治疗后PLT≥30×10^9^/L；基线PLT<20×10^9^/L时，治疗后PLT>20×10^9^/L。三系应答定义为同时发生红系、中性粒细胞和血小板应答的患者。输血依赖定义为在患者每8周输注红细胞≥2个单位。复发定义为至少连续2次外周血检测出现1系或多系血细胞计数显著或进行性下降（不能用其他临床过程解释），需要重新开始AA的治疗[17]。克隆演变定义为一种新的克隆性细胞遗传学异或出现符合MDS或AML的骨髓特征性改变[18]。

四、不良反应评价与分级

参考常见不良事件评价标准（CTCAE）5.0版进行不良反应评价与分级。

五、数据采集及随访

收集患者治疗前人口学资料、症状、体征、复核AA诊断的相关信息、输血量、血常规、血生化、EPO水平、铁代谢指标、骨髓形态、骨髓活检、染色体核型、髓系肿瘤相关基因检测等指标。通过医院电子病历与电话进行随访，中位随访时间15（6～26）个月。收集罗沙司他治疗后1、2、3、6、9、12个月症状体征，不良反应，实验室检查结果等数据；收集治疗后每6个月骨髓活检、染色体核型及相关基因指标。

六、统计学处理

采用SPSS 27.0软件进行数据描述和统计分析。分类变量组间比较采用卡方检验或Fisher精确检验。对于数值变量，首先进行正态性检验，如果各组均满足正态性，采用均数±标准差进行统计描述，采用两独立样本*t*检验进行组间比较；否则采用中位数（范围）进行统计描述，采用非参数检验进行组间比较；治疗前后实验室指标（同一个体测量2次）采用配对样本*t*检验或配对秩和检验，重复测量指标（同一个体测量>2次）采用重复测量方差分析或广义估计方程。计数资料采用百分比表示，组间比较采用非参数检验（Fisher精准检验）。双侧*P*<0.05为差异有统计学意义。

## 结果

一、基线特征

共纳入41例难治性NSAA患者，男16例（39.0％），女25例（61.0％）。其中合并PNH克隆患者11例（26.8％）。罗沙司他中位使用时间为5（3～20）个月，罗沙司他治疗前患者临床及实验室特征见表1。

**表1:** 41难治性非重型再生障碍性贫血患者罗沙司他治疗前基线特征

特征	值
年龄［岁，*M*（范围）］	52（18~84）
男/女（例）	16/25
既往治疗情况^a^［例（％）］	
EPO	32（78.0）
TPO	30（73.2）
雄激素	23（56.1）
G-CSF	10（24.4）
TPO-RA	20（48.8）
血常规［*M*（范围）］	
HGB（g/L）	79（32~107）
ANC（×10^9^/L）	1.56（0.37~4.25）
PLT（×10^9^/L）	57（1~86）
Ret（×10^9^/L）	45.7（1.9~118.7）
输血依赖［例（％）］	21（51.2）
血清铁蛋白［例（％）］	
>2 000 µg/L	4（9.6）
>1 000~2 000 µg/L	5（12.2）
≤1 000 µg/L	31（75.6）
缺失	1（2.4）
EPO水平［例（％）］	
>500 U/L	13（31.7）
≤500 U/L	25（61.0）
缺失	3（7.3）
TBIL［µmol/L，*M*（范围）］	10.2（3.0~65.6）
DBIL［µmol/L，*M*（范围）］	3.9（1.0~17.6）
ALT［U/L，*M*（范围）］	13（3~64）
AST［U/L，*M*（范围）］	26（3~92）
肌酐［µmol/L，*M*（范围）］	71（43~113）
染色体核型［例（％）］	
正常	36（87.8）
异常	1（2.4）
缺失	4（9.8）
PNH克隆［例（％）］	
1％~10％	4（9.8）
>10％~20％	5（12.2）
>20％~30％	2（4.8）

注 EPO：促红细胞生成素；TPO：促血小板生成素；TPO-RA：促血小板生成素受体激动剂；TBIL：总胆红素；DBIL：直接胆红素；PNH：阵发性睡眠性血红蛋白尿。^a^ 环孢素A除外

二、疗效评估

1. 3个月内疗效评估：所有患者经过至少3个月的罗沙司他治疗。治疗后1、2和3个月HI-E率分别为12.2％、29.2％和46.3％；其中3例（7.3％）在治疗后1个月HGB即达到正常（女性：HGB≥110 g/L，男性：HGB≥120 g/L），4例（9.8％）和6例（14.6％）分别在治疗2、3个月HGB恢复正常。在3个月获得HI-E的患者中，罗沙司他中位起效时间为2（1～3）个月。21例输血依赖的患者中，6例（28.5％）摆脱输血依赖，并同时获得HI-E，中位摆脱输血依赖的时间为3（2～3）个月。

患者在治疗前HGB为（74±20）g/L，治疗1、2和3个月后HGB分别为（79±23）、（81±25）和（85±25）g/L，采用单因素重复测量方差分析，球形检测*W*<0.001，多变量检验*F*＝3.728，*P*＝0.019，说明时间主效应（治疗1、2和3个月）HGB的变化有统计学意义。治疗1、2和3个月后分别与治疗前基线HGB进行两两比较，结果显示其差异均有统计学意义（*P*＝0.033、0.008和0.001）。

但各个时间段中性粒细胞及血小板同基线相比，无显著变化（均*P*>0.05），铁蛋白（SF）水平也无显著变化（均*P*>0.05）。

2. 6个月到随访终点疗效评估：所有患者中，罗沙司他使用时间≥6个月者19例（46.3％），其中罗沙司他使用6～<12个月者14例（34.1％），罗沙司他使用≥12个月者5例（12.2％），至本次随访结束该5例患者仍继续用药，最长持续用药20个月。

治疗后6个月，在3个月内获得HI-E的19例患者中，2例停药后复发，17例持续获得HI-E。1例患者在治疗第6个月获得HI-E，6个月的总反应率为43.9％（18/41）；其中7例（17.1％）HGB达到正常。治疗后6个月获得HI-E的患者中，罗沙司他中位起效时间为3（1～6）个月。21例输血依赖的患者中，8例（38.1％）摆脱输血依赖，并同时获得HI-E，中位摆脱输血依赖的时间为3（2～6）个月。

治疗后6个月HGB明显高于基线［（84±25）g/L对（74±20）g/L，*P*＝0.012］，但ANC、PLT、SF较基线差异均无统计学意义（均*P*>0.05）。

治疗后12个月至随访终点，仍有10例（30.3％）患者获得HI-E，其中4例HGB正常。15例输血依赖的患者中，5例（33.3％）摆脱输血依赖，并同时获得HI-E，中位摆脱输血依赖的时间为4（2～12）个月。治疗后12个月，HGB较基线明显提升［（79±19）g/L对（83±27）g/L，*P*＝0.041］，但ANC、PLT、SF较基线差异均无统计学意义（均*P*>0.05）。

患者HGB从基线至罗沙司他不同治疗时间点变化见图1。

**图1:**
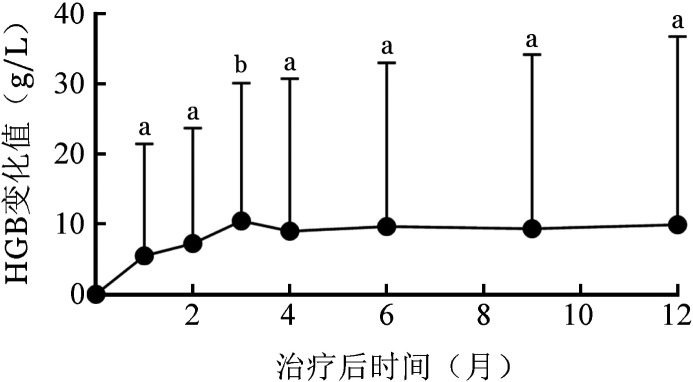
难治性非重型再生障碍性贫血患者罗沙司他治疗后血红蛋白（HGB）水平相对基线的变化趋势图（^a^*P*<0.05，^b^*P*<0.01）

三、复发和克隆演变

至随访终点，所有获得过HI-E的患者中，有9例（45％）复发，中位复发时间为7（3～12）个月。9例复发患者再次给予全剂量罗沙司他后有3例获得治疗反应，6例无效者有2例进入临床研究，1例行HSCT，其余予输血等对症支持治疗。未观察到克隆演变的发生。

四、安全性分析

9例（22.0％）患者罗沙司他治疗期间发生不良反应，最常见的不良反应为头晕（19.2％）、恶心（11.5％）、失眠（11.5％）、腹泻（11.5％）（表2）。与药物相关的不良反应均为1～2级，在对症治疗后恢复，不影响罗沙司他的使用。整个治疗期间，有1例患者死于肺部感染，与药物使用无关。

**表2:** 41难治性非重型再生障碍性贫血患者罗沙司他治疗期间不良反应发生情况

不良反应	例数（%）
头晕	5（19.2）
恶心	3（11.5）
失眠	3（11.5）
腹泻	3（11.5）
头痛	2（7.7）
肺部感染	2（7.7）
肌酐升高	2（7.7）
呕吐	1（3.8）
高血钾	1（3.8）
关节痛	1（3.8）
便秘	1（3.8）
水肿	1（3.8）

五、基线临床指标对疗效的影响

分析影响患者3个月、6个月HI-E的基线因素，结果见表3，基线年龄、性别、HGB、SF、EPO、是否为输血依赖等特征均不是影响3个月、6个月HI-E的因素。

**表3:** 影响难治性非重型再生障碍性贫血患者罗沙司他治疗获得HI-E的相关因素分析

影响因素	治疗后3个月			治疗后6个月		
	获得HI-E（19例）	未获HI-E（22例）	*P*值	获得HI-E（18例）	未获HI-E（23例）	*P*值
年龄[岁，*M*（范围）]	55（17~84）	50（16~73）	0.084	56（17~84）	50（16~73）	0.094
性别[例（%）]			0.231			0.342
男	6（31.5）	10（45.5）		6（33.3）	10（43.5）	
女	13（68.4）	12（54.5）		12（66.7）	13（56.5）	
HGB[例（%）]			0.182			0.348
30~<60 g/L	4（21.1）	8（36.4）		4（22.2）	8（34.8）	
60~90 g/L	10（52.6）	9（40.9）		10（55.6）	8（34.8）	
>90 g/L	5（26.3）	5（22.7）		4（22.2）	7（30.4）	
输血依赖[例（%）]			0.242			0.334
有	9（47.4）	12（54.5）		10（55.6）	11（47.8）	
无	10（52.6）	10（45.5）		8（44.4）	12（52.2）	
血清铁蛋白[例（%）]			0.618			0.851
≥1 000 µg/L	3（16.7）	6（27.3）		3（17.6）	6（26.1）	
<1 000 µg/L	15（83.3）	16（72.7）		14（82.4）	17（73.9）	
EPO[例（%）]			0.241			0.636
≥500 U/L	7（36.8）	6（27.3）		6（33.3）	7（30.4）	
<500 U/L	10（52.6）	15（68.2）		10（55.6）	15（65.2）	
肌酐[例（%）]			0.541			0.452
≥100 µmol/L	3（15.8）	3（13.6）		3（16.7）	3（13.0）	
<100 µmol/L	14（73.7）	17（77.3）		13（72.2）	18（78.2）	

注 HI-E：血液学改善-红系；EPO：促红细胞生成素

## 讨论

罗沙司他作为一种不良反应较低的口服药物，已被批准用于治疗接受血液透析或腹膜透析的贫血患者，并在我国上市。自罗沙司他上市以来，基于难治性NSAA庞大的患者群体及相对有限的治疗选择，其在真实世界中治疗NSAA的疗效和安全性一直在被探索，但目前国内未见报道。本研究结果显示对于既往多线治疗无效的NSAA，罗沙司他仍具有良好的有效性和安全性。

由于罗沙司他尚未被批准用于AA的治疗，本研究经过我院伦理委员会的批准（批件号：1-23PJ1532）。本研究入组患者在接受罗沙司他治疗时，均已向患者交代风险并签署知情同意书；药物剂量主要参考MDS临床试验设计（NCT03263091）及说明书；采集信息前也征求了患者的同意，并签署知情同意书。

本研究结果显示，罗沙司他治疗3个月HI-E率为46.3％，14.6％的患者在治疗后3个月HGB达正常。同时，罗沙司他对有输血依赖者的改善也非常明显，在输血依赖的患者中，28.5％治疗后摆脱输血依赖。罗沙司他起效快，HI-E中位反应时间为2（1～3）个月，输血依赖者摆脱输血依赖的中位反应时间为3（2～3）个月。这个疗效与罗沙司他治疗新诊断的低危MDS（LR-MD）的3期临床试验结果[14]和罗沙司他在治疗化疗后贫血的2期研究结果[15]接近。此外，疗效在6个月、12个月依然可以维持，HI-E率分别为43.9％，30.3％，输血依赖者也有38.1％和33.3％摆脱输血依赖。治疗各个时间点的HGB水平均较基线有明显改善，这些结果均显示了罗沙司他对于难治性NSAA的贫血有明显改善。

目前难治性AA的有效治疗药物主要为重组血小板生成素受体激动剂（TPO-RA），2012年《新英格兰医学杂志》曾报道艾曲泊帕对ATG+CsA难治性SAA的有效率为44％，但其中HI-E率仅为24％（6/25）[19]。Ruan等[20]回顾分析了41例NSAA患者（其中37例为复发/难治性NSAA），发现艾曲泊帕3个月的总有效率为51.2％，其中HI-E率为19.5％（8/41）。Jang等[21]前瞻性研究发现罗米司亭在31例复发难治AA患者（其中18例为复发/难治性NSAA）中治疗53周后三系缓解率为39％，但入组病例仅限于ATG联合CsA或CsA单药治疗无效者。一些文献报道了重组人EPO（rhEPO）在初治AA中的疗效，但并没有在复发/难治性AA患者中的报道[22]。罗特西普在PACE（NCT01749514/NCT02268383）[23]–[24]和MEDALIST（NCT02631070）[25]研究中证实了其对于伴有贫血的MDS患者的疗效，但并没有针对AA的相关研究。因此，目前缺乏针对NSAA贫血的有效治疗药物。我们的研究成果初步证实了罗沙司他的疗效，尽管为回顾性研究，但可为未来设计前瞻性研究奠定理论基础。

我们没有在血小板或中性粒细胞中观察到明显治疗反应，表明罗沙司他的促造血机制可能仅限于对贫血的改善，对粒系和巨核系均无明显作用[26]，同时罗沙司他对SF的降低亦不明显。尽管如此，本研究入组患者大多数基线SF<1 000 µg/L，且随访时间较短，可能影响结果。

至随访期末，所有20例达到过HI-E的患者中有9例（45％）复发，再次给药后有3例获得缓解，多数患者可以维持HI-E。可能因为随访期短，没有发现克隆演变。提示短期使用罗沙司他可能对AA的克隆演变影响不大。

本研究不良反应发生率为22％，所有的罗沙司他相关不良反应均为1～2级，并且在对症治疗后恢复，不影响罗沙司他的使用。其中1例患者死于肺部感染，判断与罗沙司他治疗无关。没有观察到明显的血栓事件和血管通路并发症，这与在肾性贫血中的研究结果不同[9],[12]，可能是患者治疗前无需置管等处理及血管条件不同导致的。

我们还尝试分析影响疗效的危险因素。既往的研究提示，在初治MDS患者中，EPO≥500 U/L或输血≥2个单位/8周者，对rhEPO反应差[27]；这些因素也对罗特西普治疗的效果产生影响。在我们的研究中，可能因为样本量较少，并未发现上述因素对难治性NSAA疗效的影响。罗沙司他的作用机制不同于EPO和罗特西普，EPO是在HIF的调节下在肾脏中产生的，外源性EPO给药直接解决内源性EPO产生不足的问题；罗特西普是一种重组融合蛋白，通过降低SMAD2和SMAD3信号传导，促进晚幼红细胞分化实现红系成熟[23]–[25]。而罗沙司他抑制常氧条件下HIF降解的关键酶，使HIF稳定，相关基因上调[11]。在这种情况下，罗沙司他可以使一些EPO治疗无效的患者受益。当然，我们的患者为难治性NSAA，与EPO、罗特西普研究的MDS患者存在巨大差异。由于入组的大多为经过rhEPO治疗无效或输血依赖的患者，我们的研究提示，罗沙司可能成为rhEPO难治性、或EPO基线水平较高、或输血依赖患者的挽救性治疗。其他因素如基线年龄、性别、HGB、SF等因素也不影响HI-E。

由于罗沙司他尚未获批AA适应证，本研究仅为小样本回顾性分析，存在许多局限性。但我们的初步结果显示了罗沙司他对于CsA难治的NSAA的疗效及安全性，为下一步研究提供了方向。
